# Supplementary figures and images for: International comparison of China’s digitalization level and its enlightenment
Source: PLoS One. 2024 May 16;19(5):e0303014. doi: 10.1371/journal.pone.0303014 (PMC11098332; doi:10.1371/journal.pone.0303014)

**Supporting Information**


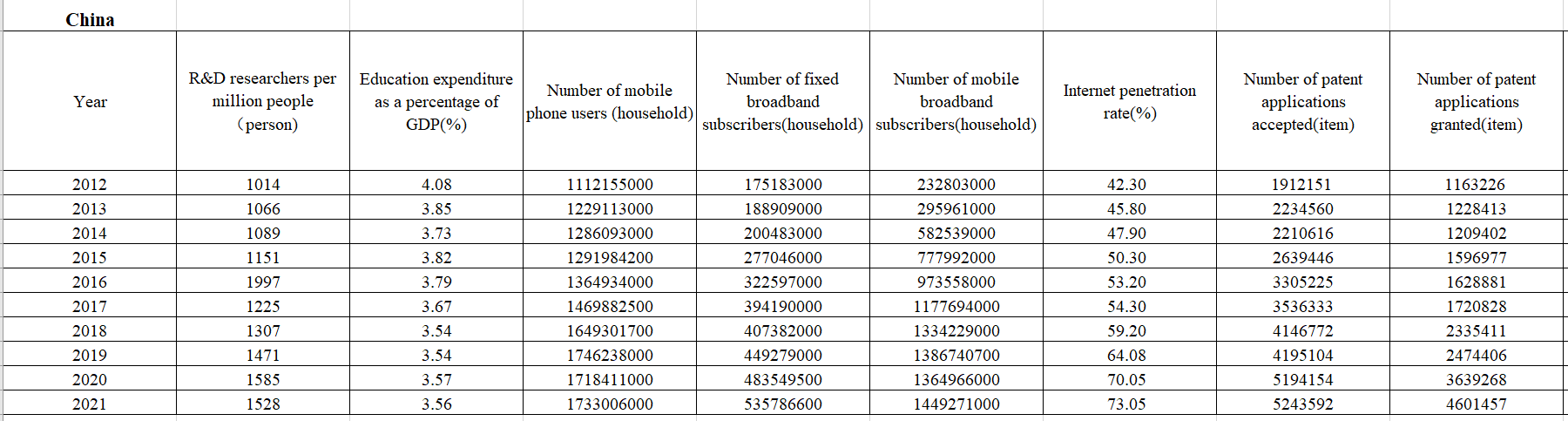


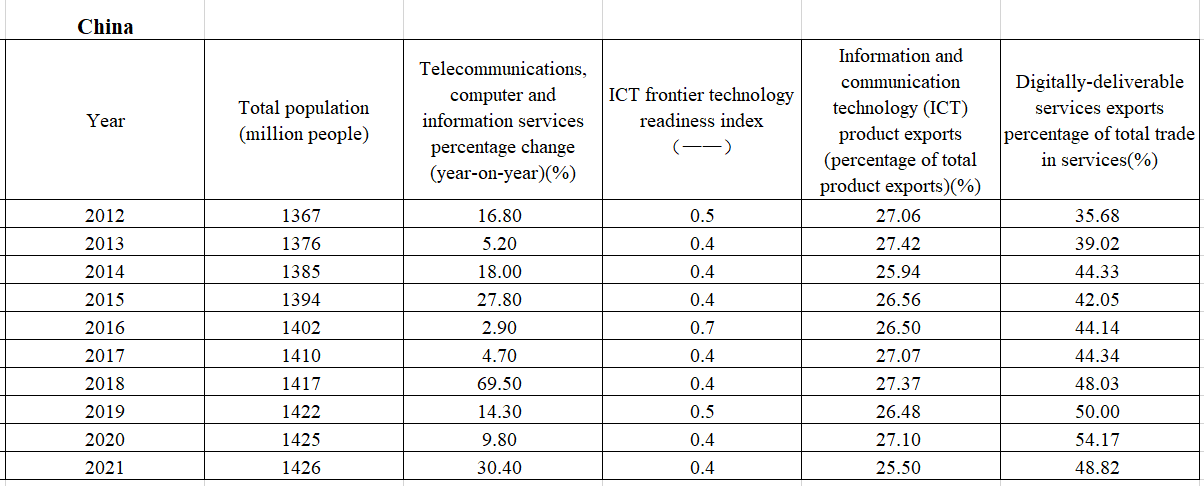


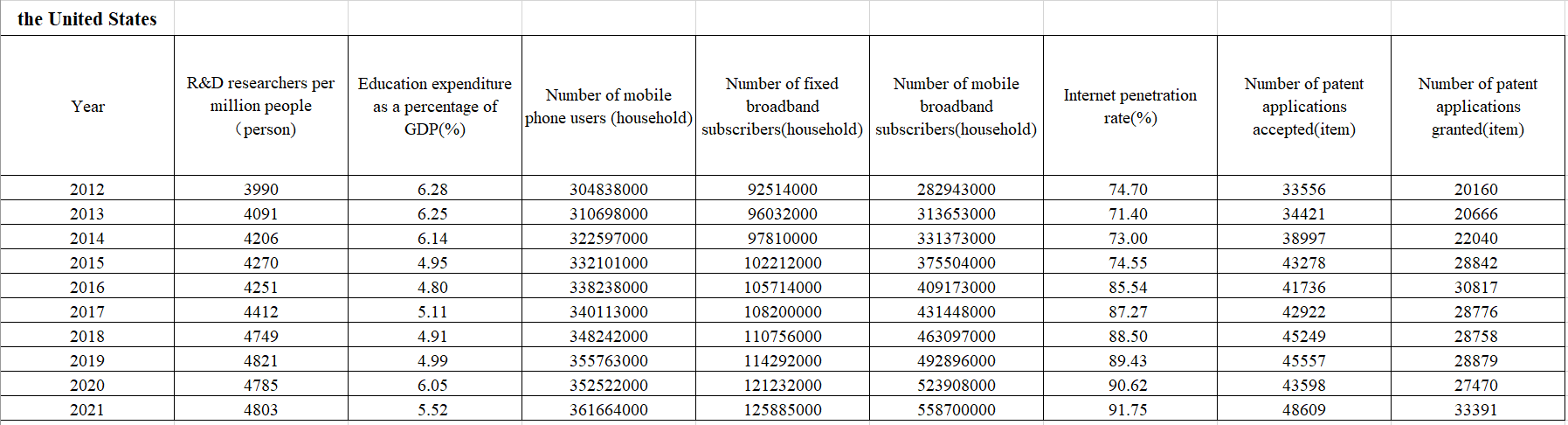


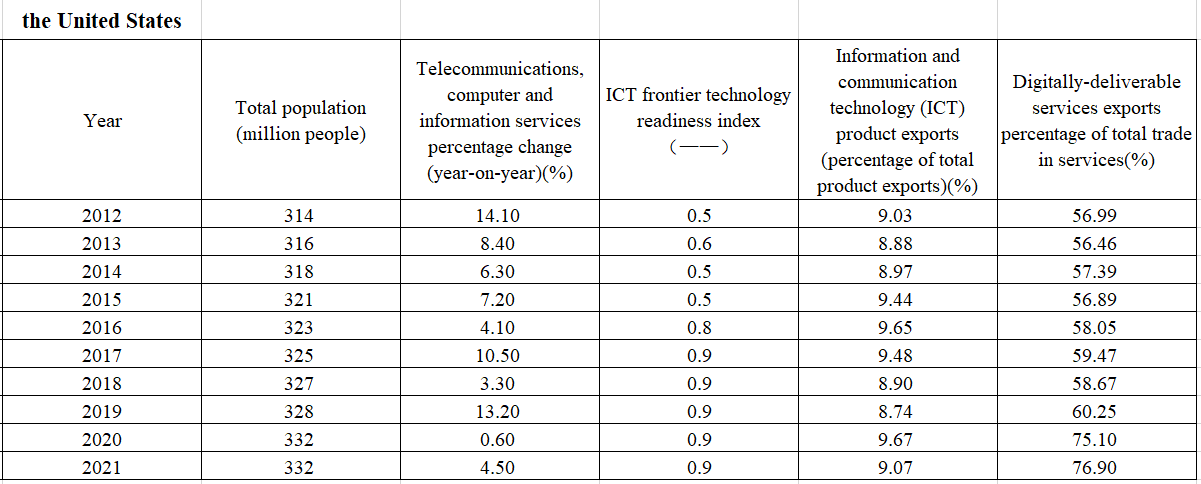


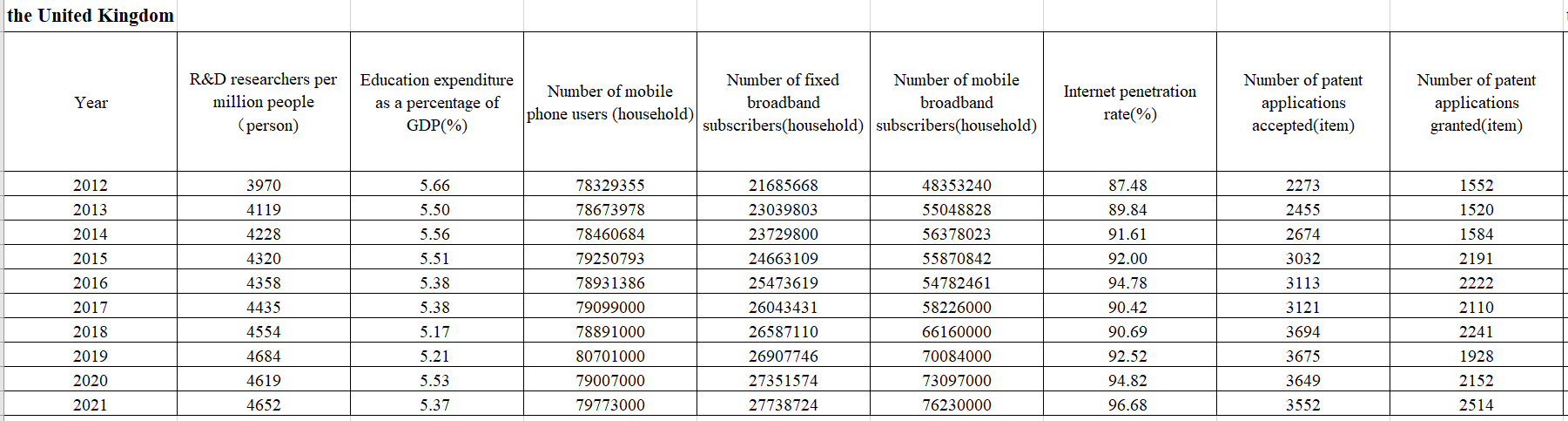


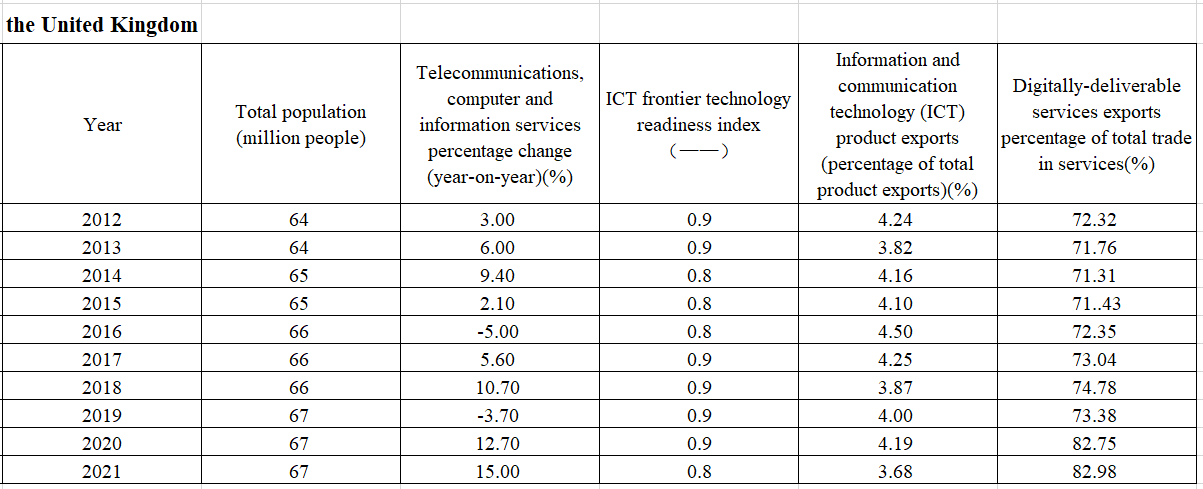


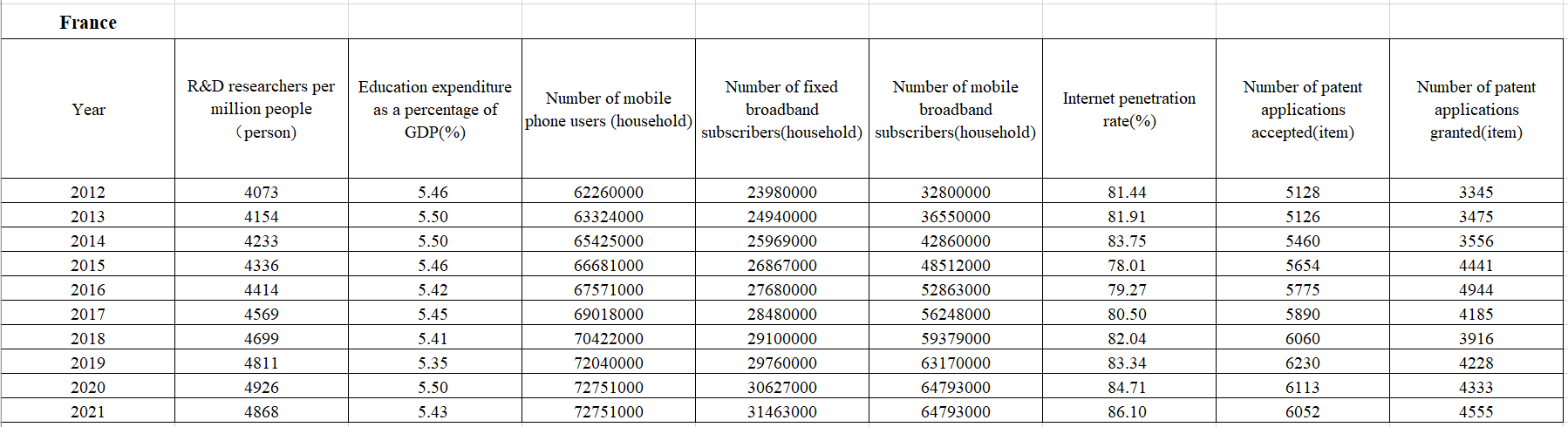


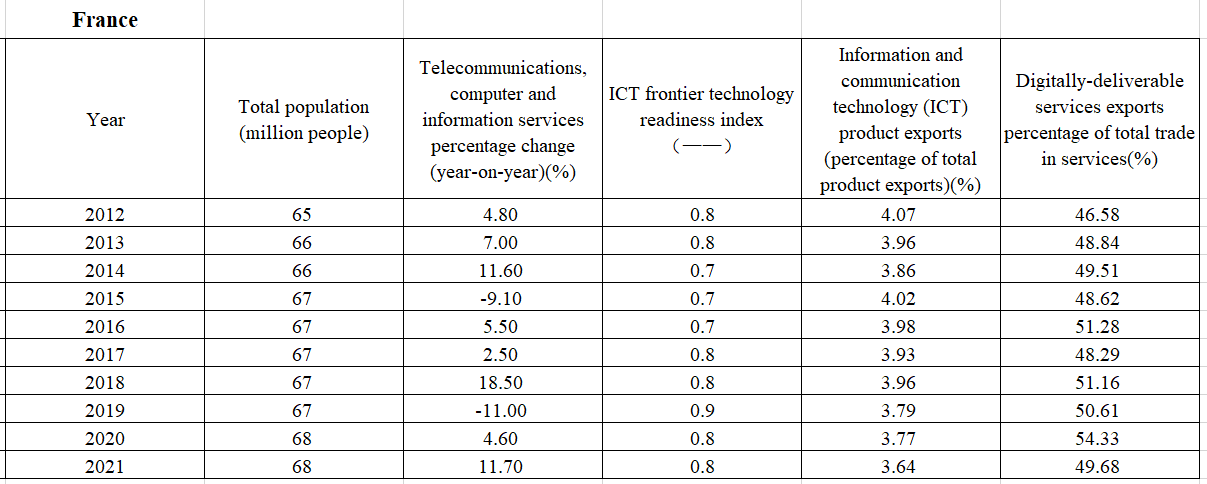


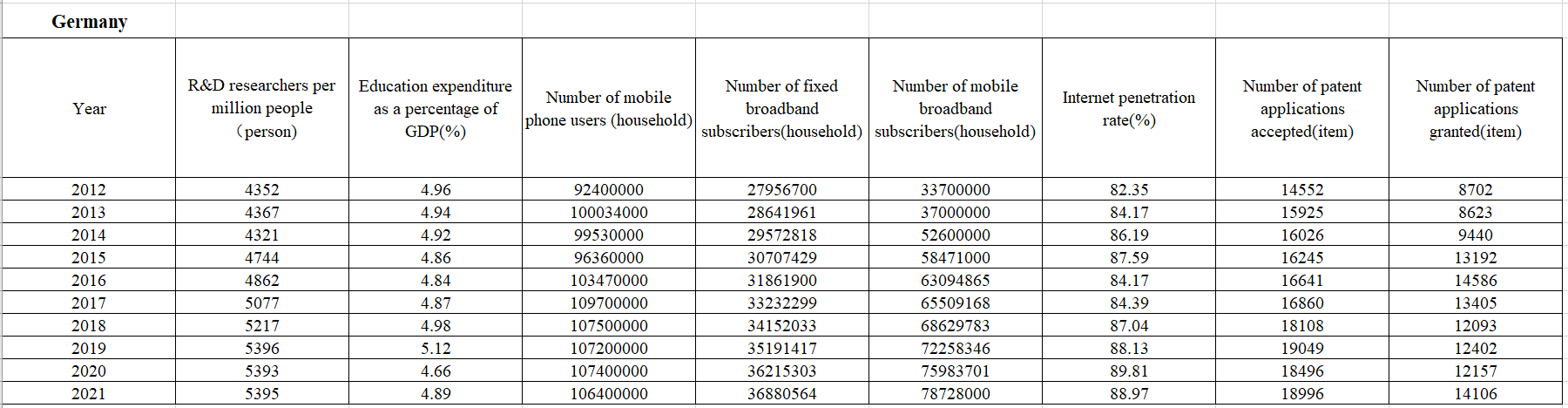


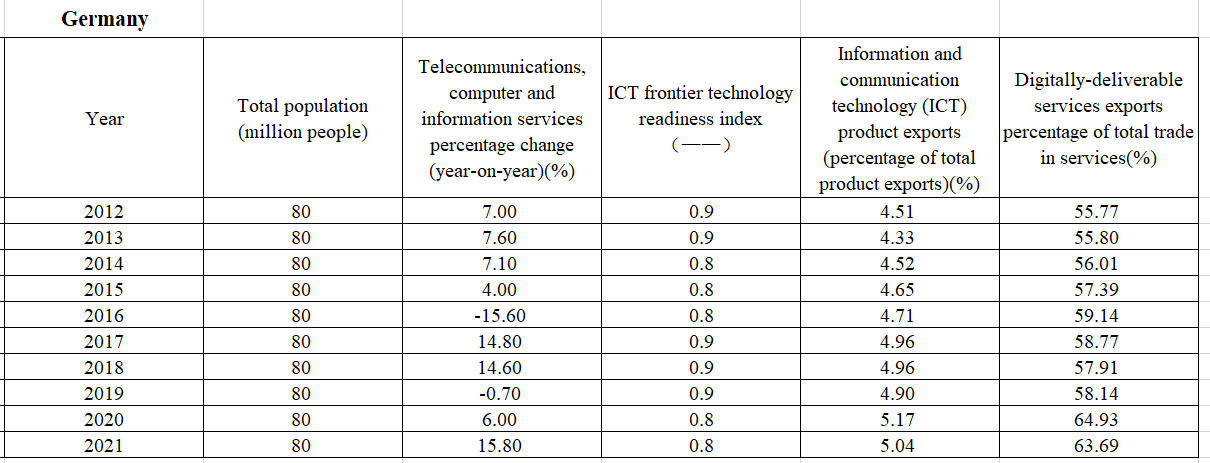


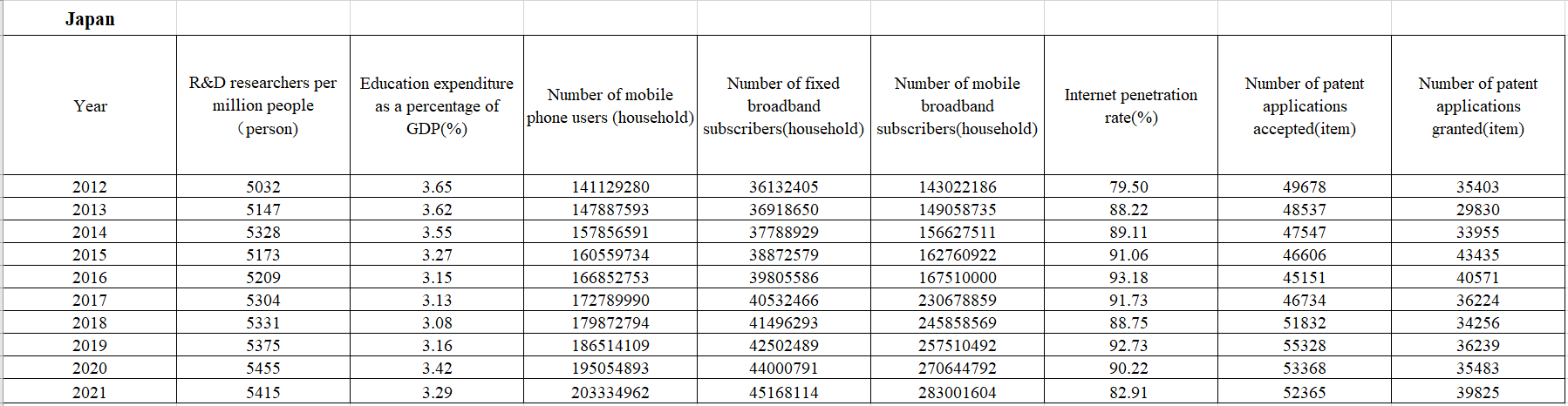


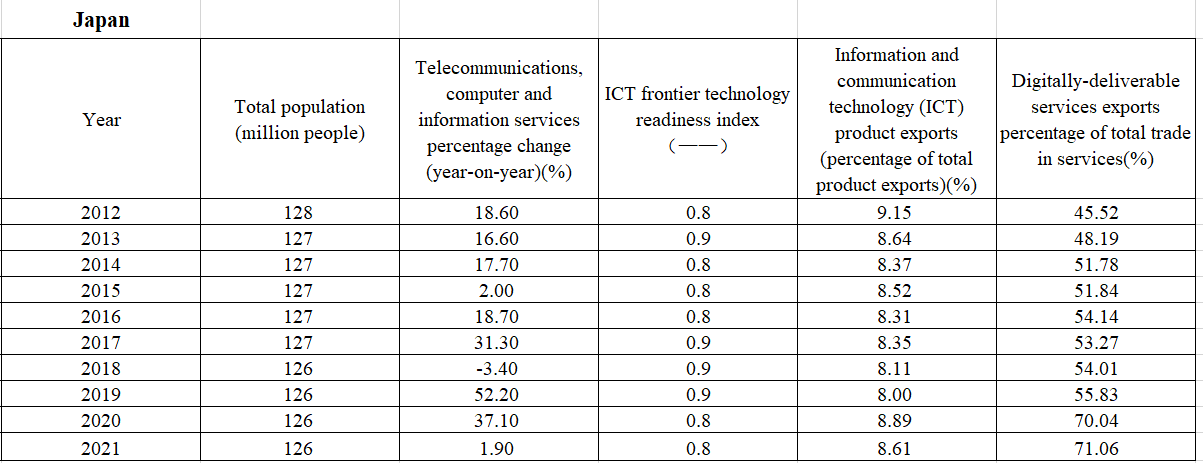


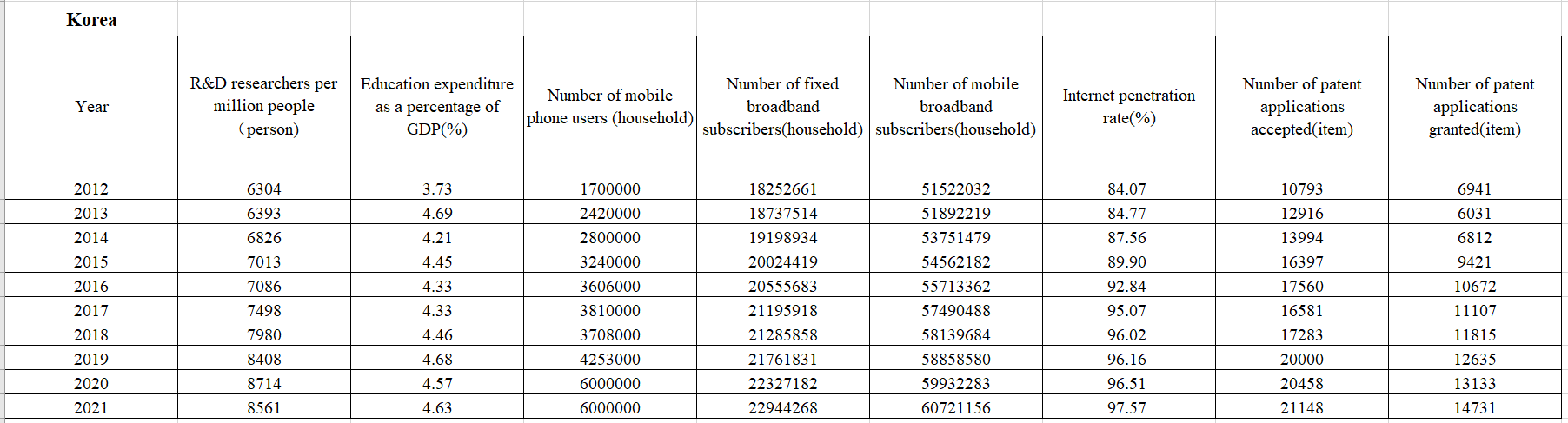


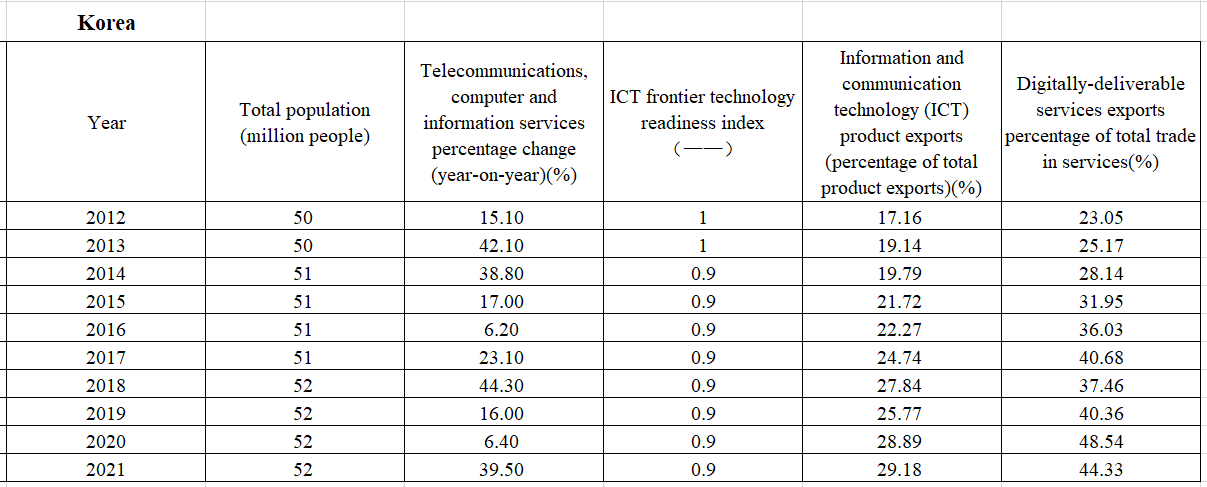

Supplement: S1 Table — (DOCX) [file pone.0303014.s001.docx]
