# Supplementary material for: International comparison of China’s digitalization level and its enlightenment
Source: PLoS One. 2024 May 16;19(5):e0303014. doi: 10.1371/journal.pone.0303014 (PMC11098332; doi:10.1371/journal.pone.0303014)
Supplement: Attachment — Submitted filename: Response to Reviewers.docx [file pone.0303014.s002.docx]

**Response to Reviewer 1 Comments**

**1. Summary**

Thank you very much for taking the time to review this manuscript. Those comments are all valuable and very helpful for revising and improving our paper, as well as the important guiding significance to our researches. We have studied comments carefully and have made correction which we hope meet with approval. Revised portion are marked in red in the paper. The responds to the reviewer's comments are as flowing:

**2. Response to author's comments**

1. Is the manuscript technically sound, and do the data support the conclusions?

The manuscript must describe a technically sound piece of scientific research with data that supports the conclusions. Experiments must have been conducted rigorously, with appropriate controls, replication, and sample sizes. The conclusions must be drawn appropriately based on the data presented.

Reviewer #1: No

Response and Revisions: According to the reviewer's comments, the conclusion part of the paper has been carefully revised. Please find the detailed responses below.

2. Has the statistical analysis been performed appropriately and rigorously?

Reviewer #1: Yes

Response to this comment: We agree with your comments.

3. Have the authors made all data underlying the findings in their manuscript fully available?

Reviewer #1: Yes

Response to this comment: We agree with your comments.

4. Is the manuscript presented in an intelligible fashion and written in standard English?

PLOS ONE does not copyedit accepted manuscripts, so the language in submitted articles must be clear, correct, and unambiguous. Any typographical or grammatical errors should be corrected at revision, so please note any specific errors here.

Reviewer #1: Yes

Response to this comment: We agree with your comments.

**3. Point-by-point response to Comments and Suggestions for Authors**

**Comments 1:** It is essential to clearly state the specific aims of the study.

**Response 1**: Thank you for pointing this out. We agree with this comment. Therefore, we have the last paragraph of the introduction to be more explicit about the specific objectives of the research.

Currently, countries around the world have launched digitalization development strategies to seize important opportunity windows. Developed countries such as Europe, the United States and Japan have accumulated long-term experience, while China is in the catch-up stage [3]. In view of this, this paper reviews and analyzes the evolution of the techno-economic paradigm in major developed countries and China, with a view to clearly understanding the gaps and shortcomings in China on the basis of learning from the advanced experience of other countries, so as to find out the effective ways and countermeasures of China's digitalization development.

This change can be found in page 3, Introduction part.

**Comments 2:** Detailed comparisons, supported by statistical data and relevant literature, are essential to draw meaningful conclusions. The conclusion section should summarize the key findings of the comparative analysis and their implications. Clearly state the contributions of your study to the existing literature. Discuss the practical implications for policymakers, businesses, and academics. Additionally, suggest avenues for future research based on the gaps identified during the comparative analysis.

**Response 2**: Agree. Therefore, we have revised our manuscript to emphasize this point.

By constructing the index system of digitalization development level, this paper objectively compares the digitalization level of our country with that of the major developed countries, reveals the gaps and shortcomings in our digitalization development, and also makes clear the position of our country in the global digitalization competition. This has a very important role in finding out the key elements to improve our country's digitalization level and help us better define the direction of development.

From the above analysis, it can be seen that there is still a large difference in the level of digitalization development between China and major developed countries, which is caused by a variety of factors. One of the direct reasons is the increasing demand for high-level digitalization human resources factors, and the insufficient match between the cultivation and demand of digitalization human resources elements. The deeper reason is that China's current techno-economic paradigm has not been able to fully adapt to the needs of digitalization development. The synergistic development of techno-economic paradigm and digitalization affects the path of digitalization development, which leads to differences in the level of digitalization development. In view of this, we need to start from the following aspects to enhance China's digitalization development level.

This change can be found in page 28-30, Conclusion part.

**Comments 3:** Ensure that each section flows logically, leading the reader from one point to the next seamlessly.

**Response 3**: Thank you for pointing this out. We agree with this comment. Therefore, we have revised the structure of the paper to emphasize this point, as noted in the red font of the paper.

**Response to Reviewer 2 Comments**

**1. Summary**

Thank you very much for taking the time to review this manuscript. Those comments are all valuable and very helpful for revising and improving our paper, as well as the important guiding significance to our researches. We have studied comments carefully and have made correction which we hope meet with approval. Revised portion are marked in red in the paper. The responds to the reviewer's comments are as flowing:

**2. Response to author's comments**

1. Is the manuscript technically sound, and do the data support the conclusions?

The manuscript must describe a technically sound piece of scientific research with data that supports the conclusions. Experiments must have been conducted rigorously, with appropriate controls, replication, and sample sizes. The conclusions must be drawn appropriately based on the data presented.

Reviewer #2: No

Response and Revisions: According to the reviewer's comments, the conclusion part of the paper has been carefully revised. Please find the detailed responses below.

2. Has the statistical analysis been performed appropriately and rigorously?

Reviewer #2: Yes

Response to this comment: We agree with your comments.

3. Have the authors made all data underlying the findings in their manuscript fully available?

Reviewer #2: Yes

Response to this comment: We agree with your comments.

4. Is the manuscript presented in an intelligible fashion and written in standard English?

PLOS ONE does not copyedit accepted manuscripts, so the language in submitted articles must be clear, correct, and unambiguous. Any typographical or grammatical errors should be corrected at revision, so please note any specific errors here.

Reviewer #2: No

Response and Revisions: We further proofread the manuscript and marked the changes in red. Thank you for your comments.

**3. Point-by-point response to Comments and Suggestions for Authors**

**Comments 1:** The purpose of the article is specified in the abstract as" this paper evaluates the digital development level of China, the United States, the United Kingdom, France, and other major developed countries in the world based on the entropy method." Then, also at the and of introduction as: "In order to better achieve this strategic goal, we need to conduct a scientific and reasonable evaluation of China's digital development level, clarify the current situation of China's digital development, and conduct a scientific comparative analysis with major leading countries in the world, to find out the effective ways and countermeasures of China's digital development. -there is a kind of discrepancy and it should be corrected.
 **Response 1**: Thank you for pointing this out. We agree with this comment. Therefore, we have revised abstract and the last sentence of the introduction.

[Abstract] In order to better measure the current status of China's digital development level, this paper evaluates the digital development level between China and the major developed countries in the world, such as the United States, the United Kingdom, France based on the entropy method, and makes a comparison and analysis between China and the major developed countries from the historical perspective of the evolution of the techno-economic paradigm. With a view to summarizing and exploring from it and drawing on the advanced experiences accumulated by the developed countries over a long period of time, so as to provide China with useful insights and bases for further enhancing its digitalization development level.

This change can be found in page 1, Abstract part.

[Introduction] In view of this, this paper reviews and analyzes the evolution of the techno-economic paradigm in major developed countries and China, with a view to clearly understanding the gaps and shortcomings in China on the basis of learning from the advanced experience of other countries, so as to find out the effective ways and countermeasures of China's digitalization development.

This change can be found in page 3, Introduction part.

**Comments 2:** The title of the point 3 is too long "3. Comparison of digital development levels between China and major developed countries based on entropy method". Moreover most of the part of it, is too much similar to the title of the article.

**Response 2:** Agree. Therefore, we have revised our manuscript to emphasize this point.

We have revised the title and the structure of the paper, with title 3 adjusted to title 4.3. "4.3 Difference analysis of digitalization development level".

**Comments 3:** So long part of the article devoted to digitalization should be presented earlier in the introduction not in the methodological part.

**Response 3:** Thank you for pointing this out. We agree with this comment. Therefore, we have adjusted the part on digitization to the first paragraph of the introduction.

**Comments 4:** Conclusion is too long and this division into subparts are unnecessary. So, most part of conclusion should be included in the results part.

**Response 4:** Agree. Therefore, we have revised our manuscript to emphasize this point. We have cut out the conclusion.

This change can be found in page 28-30, Conclusion part.

**Comments 5:** References list is limited.

**Response 5:** After the paper was revised, we rechecked the references and added references 28, 29, 52-67.
